# Correlation among job-induced stress, overall well-being, and cardiovascular risk in Italian workers of logistics and distribution

**DOI:** 10.3389/fpubh.2024.1358212

**Published:** 2024-04-09

**Authors:** Santo Fruscione, Ginevra Malta, Maria Gabriella Verso, Anna Calascibetta, Daniela Martorana, Emanuele Cannizzaro

**Affiliations:** ^1^PROMISE Department, University of Palermo, Palermo, Italy; ^2^Department of Orthopedics, Hospital Company ‘Ospedali Riuniti Villa Sofia-Cervello’, Palermo, Italy

**Keywords:** occupational medicine and hygiene, risk, job, cardiovascular prevention, occupational health, prevention

## Abstract

**Introduction:**

Work-related stress is an occupational risk that has been linked to the development of cardiovascular disease (CVD). While previous studies have explored this association in various work contexts, none have focused specifically on logistics and distribution personnel. These workers may be exposed to significant job stress, which potentially increases the risk of CVD.

**Methods:**

In this study, we aimed to examine the relationship between work-related stress and cardiovascular risk in a sample of 413 healthy workers of a logistics and distribution company. To assess work-related stress and cardiovascular risk, we used the organisational well-being questionnaire proposed by the Italian National Anti-Corruption Authority, the Framingham Heart Study General Cardiovascular Disease (CVD) Risk Prediction Score and the WHO General Wellbeing Index (WHO-5).

**Results:**

Our results revealed that individuals with low job support had a significantly higher CVD risk score and lower well-being index than those reporting high job support. Furthermore, workers with high-stress tasks showed higher well-being index scores than those with passive tasks. Approximately 58% of the subjects were classified as low CVD risk (CVD risk <10%), approximately 31% were classified as moderate risk (CVD risk between 10 and 20%) and 11% were considered high risk (CVD risk >20%). The overall median CVD risk for the population was moderate (6.9%), with individual scores ranging from 1 to 58%.

**Discussion:**

Further analyses confirmed the protective effect of work support, also identifying physical inactivity, regular alcohol consumption and low educational level as factors contributing to an increased risk of CVD. Interestingly, factors such as job control and work support demonstrated a positive impact on psychological well-being. These results emphasise the importance of intervention strategies aimed at promoting health in the workplace. By addressing these combined factors, organisations can effectively reduce the risk of CVD and improve the general well-being of their workforce.

## 1 Introduction

Cardiovascular diseases (CVD) occurring in adulthood are mainly caused by a combination of modifiable and non-modifiable factors (1). These diseases are the leading cause of death in developed countries (2).

Among the factors that can be changed, behaviours such as smoking, unhealthy eating habits, lack of physical activity and stress have been identified as significant factors (3, 4). However, although there is international consensus that work-related stress is a major health and safety challenge in modern society, its connection to cardiovascular risk is not yet fully understood. With the ageing of the world’s population and considering its particular impact on certain occupations, it is expected that an increasing number of workers will develop chronic diseases during their working lives, with a significant impact on essential services for the general population (5).

In recent years, also due to the SARS-CoV-2 pandemic with increased sedentary behaviour, workplace health promotion has become a crucial issue on an international scale (6, 7). In Italy, a recent agreement between the Italian Society of Occupational Medicine (SIML) and the Ministry of Health emphasises the role of occupational physicians in the implementation of strategies for the prevention of chronic non-communicable diseases among workers and citizens (8). These strategies include the implementation of screening and the promotion of healthier lifestyles. To ensure the effectiveness of these strategies, it is important to first explore the relationships between organisational factors, psychological well-being and general health status in different work environments. It is precisely as a result of the COVID-19 pandemic and its variants that the concept of psychological well-being has also been reinforced in relation to the work dimension (9). In fact, psychological stress was able to lead to erroneous behaviour and addictions, such as those to alcohol, which could also negatively influence the individual’s working life (10).

It will be necessary to identify the groups of workers who may be more susceptible to health problems and to design specific workplace health promotion programmes (11). Despite the well-established association between work-related stress and cardiovascular risk factors such as metabolic syndrome or hypertension, very few studies have examined this association in specific occupations (12–14). To our knowledge, few studies have been conducted on a sector as heterogeneous and varied as logistics and distribution, which has experienced an increase in the demand for labour in recent years. This complex system involves a large number of people, from drivers to warehousemen and administrative staff. Managing this workforce, ensuring their well-being and optimising their performance is a significant operational challenge. In summary, the logistics and distribution sector is a very intricate web of processes, technologies, regulations and human resources (15, 16). It is constantly evolving to meet the needs of a rapidly changing world and the associated complexities require constant innovation and adaptation to ensure timely and reliable delivery of items to individuals and businesses.

To assess work-related stress, validated methods use self-administered questionnaires such as the Effort/Reward Imbalance (ERI) and the Job Demand-Control (JDC), which are highly reliable (16, 17). In particular, Karasek’s job demand-control model, although proposed in 1979, remains relevant today, using a system that identifies job demand and job control as key risk factors for employee well-being (18). Work demand refers to quantitative aspects such as workload and time pressure, while work control, or ‘decision latitude’, refers to workers’ ability to control their workload and schedules. Combining these two dimensions, Karasek classified jobs into four categories: high-stress jobs (high demand and low control), low-stress jobs (low demand and high control), passive jobs (low demand and low control) and active jobs (high demand and high control) (19). The JDC model was further extended to include job support as a third component. According to the theorisation of the model, jobs characterised by high demands, low control and low social support are the most detrimental to workers’ well-being (20). In our study, we used a cross-sectional design to investigate the relationship between work-related stress, employees’ general perception of emotional well-being and cardiovascular risk among workers with different tasks in a logistic and distribution company.

A secondary objective was to identify indicators of the effectiveness of health promotion programmes aimed at encouraging healthy lifestyles in this specific organisational context.

## 2 Materials and methods

During the annual occupational health surveillance programme conducted in 2023, our study focused on the recruitment of workers in a logistics and distribution company of southern Italy (21).

**Figure 1:**
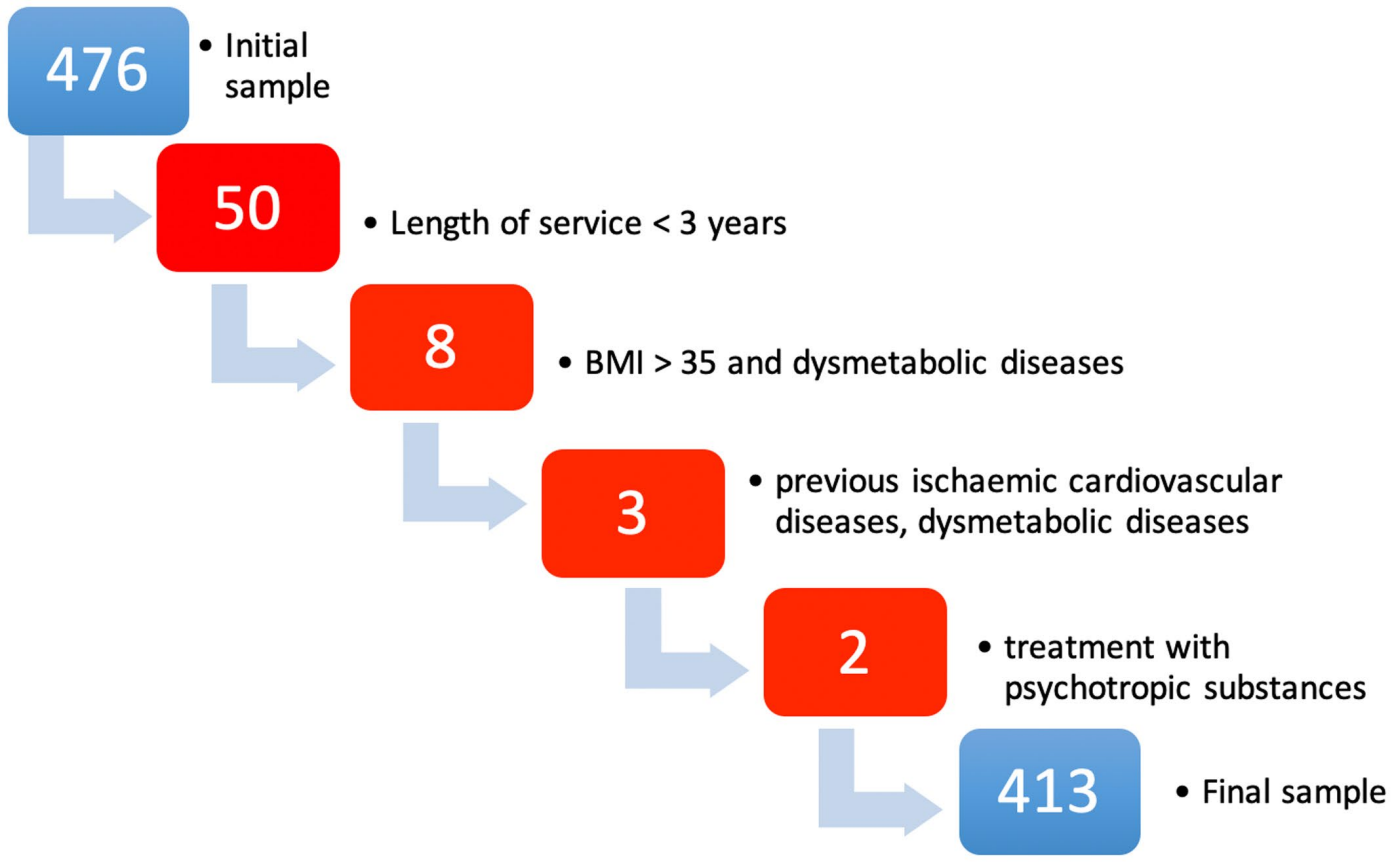
Flowchart about sample selection according to exclusion criteria.

Out of a total of over 1,000 employees, we successfully recruited 413 workers as explicated in Figure 1. The selection of subjects included those who were to undergo the occupational health surveillance programme during the study period from January 2023 to September 2023. Inclusion criteria included: an age between 18 and 65 years and at least 3 years of employment. The exclusion criteria were: previous ischaemic cardiovascular diseases, dysmetabolic diseases, Body Mass Index (BMI) > 35, treatment with psychotropic substances (22). The exclusion criteria are specifically designed to ensure that the study accurately assesses the impact of work-related stress on cardiovascular disease risk. By excluding individuals with previous ischemic cardiovascular diseases, the study aims to isolate the effects of work stress from pre-existing cardiovascular conditions. Similarly, excluding those with dysmetabolic diseases and severe obesity addresses confounders that independently increase cardiovascular disease risk, ensuring the focus remains on how work stress contributes to new cardiovascular issues. Excluding participants on psychotropic substances further clarifies this relationship by removing the potential influence of medications on heart health. This strategic selection enhances the study’s precision in investigating how occupational stress uniquely affects cardiovascular disease risk among logistics and distribution workers.

Figure 1 shows the exclusion criteria and the number of persons excluded for each cause.

During the clinical examination, eligible subjects were informed about the objectives of the study and asked to provide written consent for participation. The logistics and distribution company operates throughout the province of Palermo, carrying out most of its receiving and sorting activities within a single facility. The workers were divided into two work shifts: fixed day shifts (reference group consisting of staff with predominantly administrative tasks) and variable shifts including night shifts (reference group consisting of production, in-house processing and drivers).

Various data were extracted from the medical records of each study subject, including age, education level, job duties, length of employment, work shifts, BMI, resting heart rate, systolic and diastolic blood pressure, medical history and current drug treatments. Systolic blood pressure (SBP) and diastolic blood pressure (DBP) were measured by an experienced physician using a manual sphygmomanometer after the subjects had remained supine for 2 min making sure that they do not having consumed coffee or smoked cigarettes for at least the last 2 h before the measurement. We defined obesity as subjects with a BMI greater than 30 kg/m2 and set the threshold for hypertension at SBP ≥ 140 mmHg or DBP ≥ 90 mmHg (22, 23). Each subject also completed a standardised self-administered questionnaire, which assessed work-related stress and included information on lifestyle habits such as smoking, alcohol intake, recreational physical activity and coffee consumption.

We classified the lifestyle, personal and professional variables as follows:

- Smoking habits: Current vs. former smokers or individuals who have never smoked.
- Recreational physical activity: regular exercisers, defined as those who engage in physical activity at least twice a week, as opposed to sporadic exercisers (less than twice a week) or non-practitioners.
- Alcohol intake: abstinent (who strictly abstain from drink), sporadic drinkers (who consume less than one alcoholic unit per day, as in the case of social drinking), daily drinkers (who consume at least one alcoholic unit per day).
- Coffee intake: low consumers (one or no cup per day), medium consumers (two to four cups per day) and high consumers (more than four cups per day).
- Type of shift work: Permanent daytime (who works only day time, morning or afternoon, for 6 h) and shift workers (who works nighttime, for 12 h).
- Education: lower education (primary schools), middle education (secondary schools) and higher education (bachelor’s, master’s and doctoral degrees).
- Tasks: administrative staff, call centre operators, production staff, distribution staff.

### 2.1 Stress assessment and occupational well-being

All participants in the study completed a self-administered questionnaire that included the questionnaire on organisational well-being proposed by the Italian National Anti-Corruption Authority (24). The questionnaire consists of several subscales, including the part on organisational wellbeing, the degree of sharing of the evaluation system and the evaluation of the hierarchical superior. To assess the reliability of each subscale, Cronbach’s α values were calculated, which were 0.81, 0.68 and 0.73, respectively, for organisational well-being, the degree of sharing of the evaluation system and the evaluation of the hierarchical superior (25).

Karasek’s taxonomy of jobs was used, which classifies various jobs into four different categories: passive jobs (low demands and low job control), low-tension jobs (low demands and high job control), high-tension jobs (high demands and low job control) and active jobs (high demands and high job control) (18). This classification is based on the combination of the demand and job control scores, comparing them with the median values in a two-by-two matrix. In addition, we classified the four categories of the work taxonomy according to high or low support from colleagues. Work support scores above or below the median were used to define high and low work support, respectively.

To measure the well-being of the study participants, we used the WHO-Five Well-Being Index (WHO-5), consisting of five items (26). These items assess feelings of cheerfulness, good mood, calmness, relaxation, vigour, freshness when waking up and engagement in daily activities. Participants rated each item on a 5-point scale ranging from 0 (never) to 5 (always), indicating the perceived frequency of experiencing each item over the past 2 weeks. The raw score was obtained by summing the individual scores for the five items. The theoretical range of the raw score is from 0 (indicating no well-being) to 25 (indicating maximum well-being). The internal reliability of this well-being subscale, as assessed by Cronbach’s α, was 0.84.

### 2.2 Cardiovascular risk assessment

We used the individual SCORE2 score calculation to calculate the absolute risk of CVD, expressed as a percentage probability, for each study participant (27). This score takes into account various concomitant risk factors, including gender, age, diabetes, smoking, systolic blood pressure, total cholesterol, and continent. The 10-year risk estimate provided by this model represents the probability of developing a significant CVD event, such as coronary death, myocardial infarction, coronary insufficiency, angina, ischaemic stroke, haemorrhagic stroke, transient ischaemic attack, peripheral artery disease or heart failure (28). It should be noted that this particular model is specifically applicable to individuals aged between 40 and 89 years who did not have a pre-existing diagnosis of CVD at baseline clinical examination (29).

It should be noted that individuals under 40 years of age are by definition identified as being at low cardiovascular risk unless there are congenital malformations or diseases (27).

Since blood lipid levels are not part of routine workplace health surveillance, we chose to use BMI instead, as recommended for the simplified Framingham Study risk scoring model BMI was calculated, based on the data declared in the anamnestic section of the questionnaire, according to which, by dividing the weight expressed in kilogrammes (kg) with the height expressed in metres squared, a value is obtained that indicates the extent of body weight, distinguishing the following conditions: severely underweight (BMI < 16.5), underweight (BMI between 16.00 and 18.49), normal weight (BMI between 18.5 and 24.99), overweight (BMI between 25.00 and 29.99), mild obesity—class I (BMI between 30.00 and 34.99), medium obesity—class II (BMI between 35.00 and 39.99), severe obesity—class III (BMI > 40.00) (22, 28). The CVD risk was then classified as low (<10%), moderate (10–20%) or high (>20%).

### 2.3 Statistical analysis

To analyse the data, we used various statistical techniques, including parametric and non-parametric descriptive techniques. To compare differences between the study groups, we used analysis of variance (ANOVA) or the Kruskal-Wallis test and Spearman correlation was used analysing the relationship between variables (30, 31).

To explore the relationship between work-related stress and the risk of CVD, we constructed a multiple regression model (32). This model considered various factors, such as job demands, job control and job support scores, while adjusting for education, alcohol intake, recreational physical activity and DBP. Furthermore, we used a linear regression model to predict the well-being index, taking into account work-related stress scores together with age, duration of employment, level of education, alcohol intake, smoking habit, BMI, SBP and DBP. In another regression model, we examined the predictive value of Karasek’s categories and job support, instead of stress scores, for both CVD risk and well-being. In this analysis, we also included shift time as an independent variable.

Statistical analysis was performed with the RStudio software (33). It was not necessary to receive confirmation from the Ethical Committee as the activity is ruled by the Law Decree (DL) 81/08 within the health promotion actions. However, all participants were fully informed about the study and written informed consent was obtained from those who chose to participate. The questionnaires were anonymous, coded and securely stored by the principal investigator (MC), who was the only person authorised to access them. The questionnaires were kept separate from the clinical records of each participant. The study complied with the principles of the Declaration of Helsinki (34).

## 3 Results

Table 1 provides an overview of the main characteristics of the study participants.

**Table 1:** The study population encompassed 413 participants, and data were accessible for all selected variables.

Parametric variables	Min.	Max	Mean	Sd
Year of employment	3	41	18,47	10,72
Weight	46	112	75,65	18,08
Height	152	192	169	11,24
SBP	90	165	117,7	17,8
DBP	50	100	74,3	12,77
Heart rate	52	112	80	15,92
Non-parametric scores	Median		Interquartile range	
WHO-5- index	13		10	16
CVD risk	7,5		5,0	12,43
Job Demand Score	14		11	17
Job Control Score	23		20	26
Job Support Score	16		14	17
Categorical variables				
Coffee	Low		112	27
	Medium		240	58
	High		61	15
Level of institution	Low		57	14
	Medium		247	60
	High		109	26
Working shifts	6 h		275	66
	12 h		138	34
CVD risk class	Low < 10%		237	58
	Medium 10–20%		127	31
	High > 20%		49	11
Job description	Call centres		42	10
	Postmen		116	28
	Employee		58	14
	Production clerk		197	48

Among the 413 participants, 234 were male (57%) and 179 (35) female, the mean age was 43.27 years (standard deviation [sd] 13.23) and the mean BMI was 26.18 (sd 4.62).

Approximately 30% of the study population were habitual smokers, while 59% were classified as overweight. In addition, 16% of the participants regularly drank alcohol and 67% regularly engaged in recreational physical activity. It was found that 24.6% of the participants suffered from hypertension.

**Figure 2:**
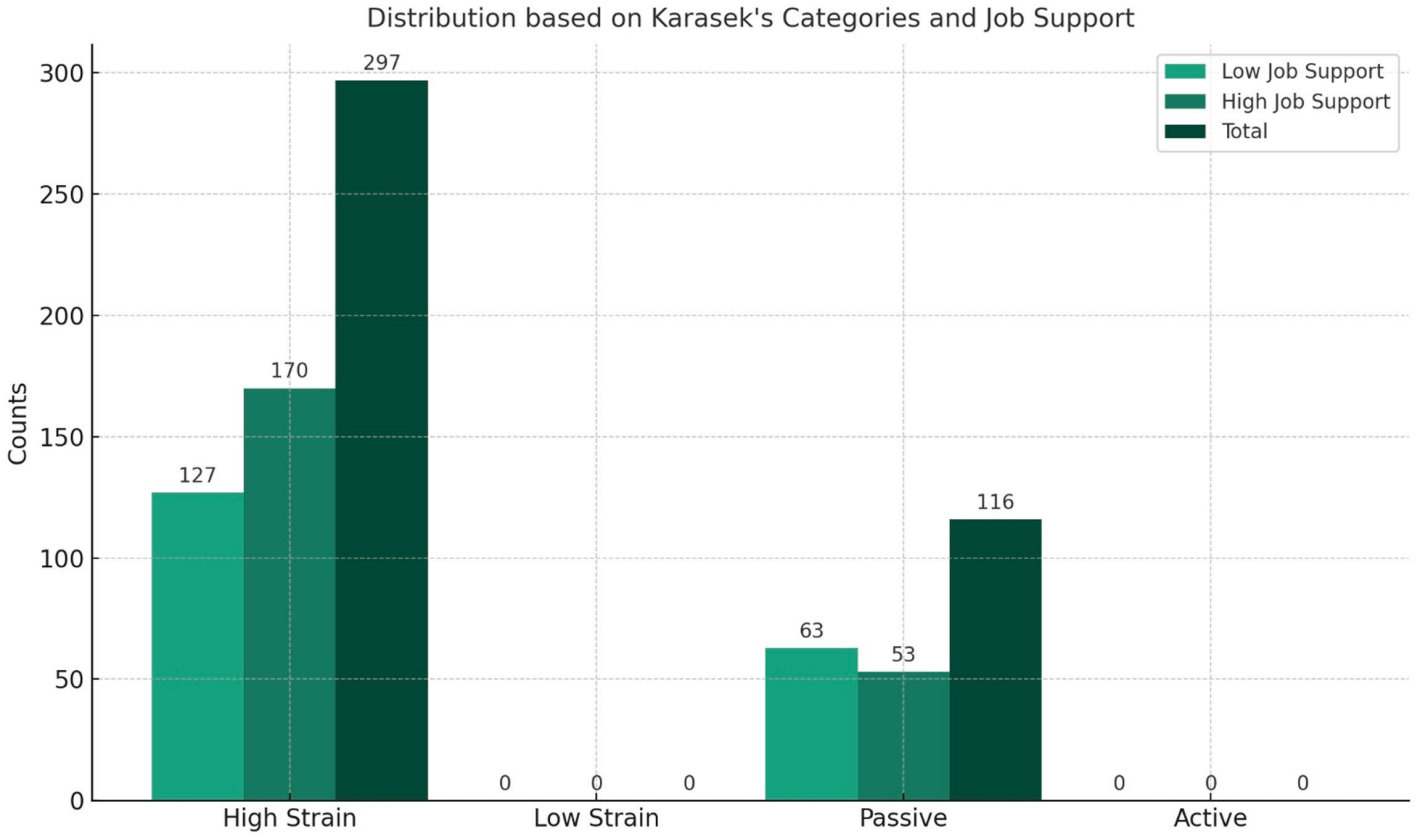
The overall study population’s distribution based on Karasek’s categories and Job support categories.

Figure 2 presents the distribution of the study subjects according to Karasek’s categories and job support categories. Passive jobs were less common, accounting cumulatively (call centres and office workers) for 28% of the sample, with none of the jobs falling into the low-voltage categories. Karasek’s categories were further divided according to job support, with 46% falling into the low-support category and 54% into the high-support category. The cut-off points for the work demand, control and support scores according to Karasek’s categorisation were 13, 24 and 15, respectively.

The subjects in the different Karasek and work support categories had similar characteristics in terms of age, duration of employment, BMI, SBP, DBP and heart rate (HR; not shown in the tables).

The study population was classified into different CVD risk levels in Table 2. Approximately 58% of the subjects were classified as low CVD risk (CVD risk <10%), approximately 31% were classified as moderate risk (CVD risk between 10 and 20%) and 11% were considered high risk (CVD risk >20%). The overall median CVD risk for the population was moderate (6.9%), with individual scores ranging from 1 to 58% (30).

**Table 2:** Results from the Kruskal-Wallis test comparing CVD risk and WHO-5 among Karasek’s categories and Job support categories in the overall study population.

	Passive		Low strain		Active		High strain	Kruskal-Wallis	
	*N* = 116 (28%)		*N* = 0		*N* = 0		*N* = 297 (72%)		*p*
	Med	IQR	Med	IQR	Med.	IQR	Med.	IQR	
CVD risk Score	7.3	4.32–10.52	-	-	-	-	8.3	5.5–12.0	0.435
WHO-5	13.0	10.0–15.0	-	-	-	-	14	11.0–18.0	0.005
		Low support				High support			
		*N* = 190				*N* = 223			
		Med	IQR			Med	IQR		
CVD risk Score		8.2	5.25–12.2			7.5	4.6–12.8		0.043
WHO-5		12	9.0–15.0			16	13–19		<0.001

The passive job category showed lower values in the WHO well-being index than high-stress jobs (*p* = 0.005), as observed in Table 2.

**Figure 3:**
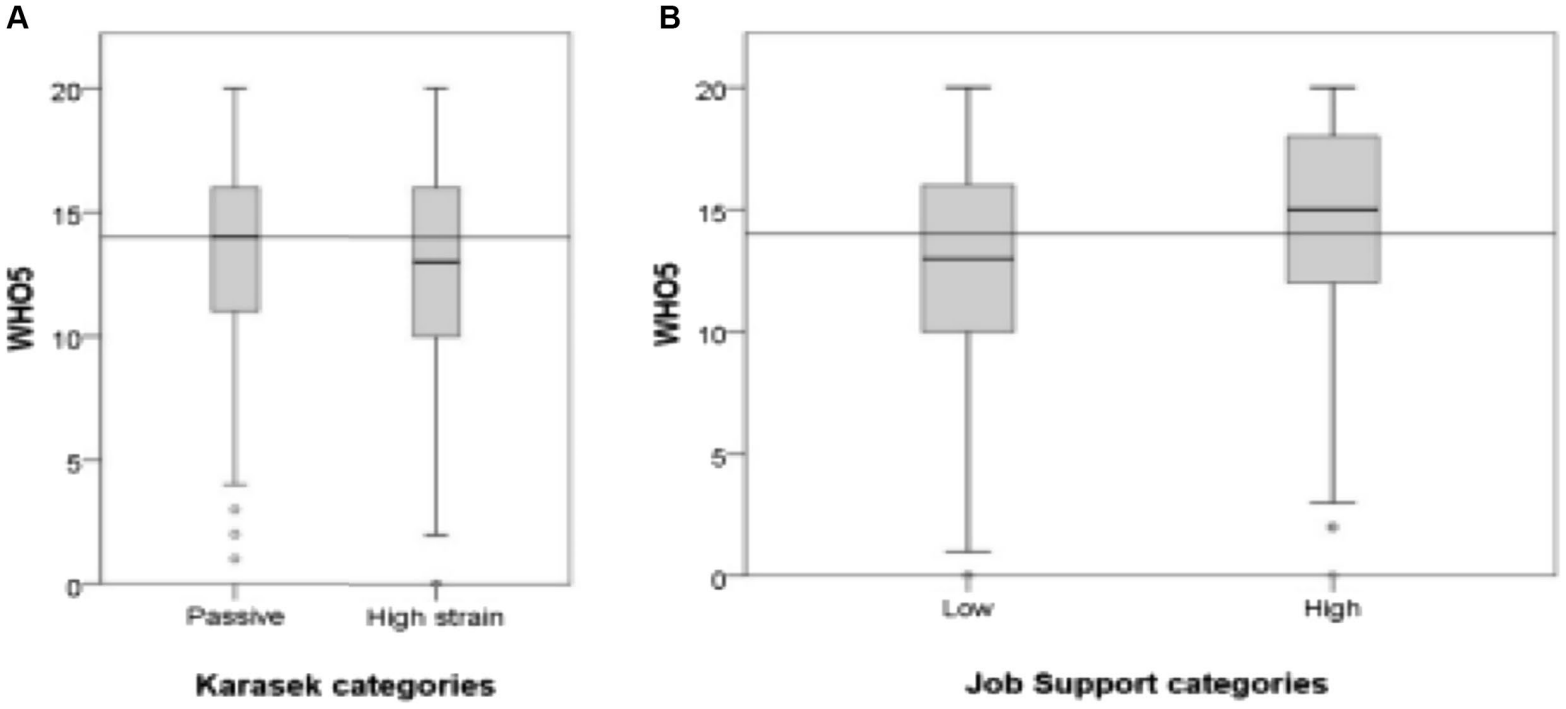
Primary median (depicted by the grey line) and box plots illustrating general well-being (WHO-5): within the four Karasek’s categories **(A)**; and across job support categories **(B)**.

The average CVD risk score did not exceed the low threshold in either of Karasek’s two categories in our sample. A low level of work support was moderately, but significantly associated with increased CVD risk scores and lower WHO well-being index, compared to high work support (Table 2; Figure 3).

The correlation matrix shown in Table 3 indicates that the CVD risk score was positively correlated with regular alcohol consumption (*p* = 0.023) and higher resting heart rate (*p* < 0.001), and negatively correlated with leisure-time physical activity (*p* ≤ 0.001), well-being index (*p* ≤ 0.001) and work support score (*p* = 0.037). In contrast, the WHO-5 well-being index was positively correlated with job control (*p* < 0.001) and job support (*p* < 0.001), and negatively correlated with job demand (*p* = 0.047).

**Table 3:** Spearman correlation matrix (*p < 0.05; **p < 0.01).

					*p < 0.05, **p < 0.01							
	CV RISK score	Educational level	Duration of employment	Alcohol	Coffee	Physical activity	WHO-5	Heart rate	Demand score	Control score	Support score	DBP
CVD RISK score	1.000	−0,286**	0.061	0.096*	0.029	−0.183**	−0.217**	0.211**	0.038	0.044	−0.088*	0.415**
Education Level		1.000	−0.115**	−0.059	−0.013	0.071	0.047	−0.121**	−0.014	0.032	0.065	−0.1*
Duration of employment			1.000	0.033	0.016	0.011	0.002	0.024	0.097*	0.035	0.04	−0.018
Alcohol				1.000	0.021	−0.044	−0.043	−0.006	−0.024	−0.002	−0.06	0.071
Coffee					1.000	−0.022	−0.047	−0.015	0.002	−0.011	−0.091*	−0.11**
Physical activity						1.000	0.089*	−0.253**	−0.027	−0.021	−0.054	−0.112**
WHO-5							1.000	−0.022	−0.083*	0.162**	0.319**	−0.032
Heart Rate								1.000	0.01	−0.045	0.053	0.198**
Demand score									1.000	−0.232**	−0.235**	0.071
Control score										1.000	0.321**	−0.021
Support Score											1.000	−0.051
DBP												1.000

DBP showed a strong correlation with SBP (Pearson’s correlation coefficient = 0.702, *p* < 0.001, not shown in Table 3) and a moderate correlation with CVD risk (Table 3).

The linear regression model for predicting CVD risk showed a negative effect of systolic blood pressure, low education level and physical inactivity, which tended to increase the risk of CVD. In contrast, a higher work support score protected against CVD risk, as did abstention or occasional consumption of alcoholic beverages (Table 4). The median values of the expected CVD risk, classified according to work support and Karasek categories, are presented in Table 5. When Karasek’s categories were entered instead of job application, control and support scores in the regression model for predicting CVD risk, no significant impact on CVD risk was observed (R2 = 0.279; adjusted R2 = 0.265).

**Table 4:** Table summarising the Relative Risks (RR) and 95% Confidence Intervals for the variables considered.

Variables	Relative risk (RR)	95% CI lower bound	95% CI upper bound
Intercept	4.36e-06	4.24e-09	4.48e-03
Job Demand Score	0.994	0.891	1.110
Job Control Score	1.001	0.883	1.135
Job Support Score	0.818	0.680	0.985
Education Low	21.55	1.08	430.18
Education Medium	0.839	0.044	16.12
Alcohol Abstinent	0.210	0.065	0.682
Alcohol Weekend	0.107	0.026	0.447
Physical Activity No	7.83	2.81	21.86
DBP	1.357	1.276	1.443

The independent variables include educational level, alcohol intake, DBP, continuous job demand score, continuous job control score, continuous job support score, and physical activity.

**Table 5:** Multiple linear regression model forecasting WHO-5 well-being (R2 = 0.1661; adjusted R2 = 0.1447).

Variable	Relative risk (RR)	95% CI
Intercept	10170.41	57.01–1814435.79
Job demand Score	1.017	0.946–1.093
Job control Score	1.126	1.037–1.222
Job support Score	1.420	1.259–1.602
Age	0.865	0.820–0.914
Duration of Employment	0.982	0.938–1.027
BMI	0.978	0.869–1.100
SBP	1.009	0.975–1.043
DBP	1.024	0.968–1.084
Education Low	1.020	0.137–7.602
Education Medium	0.697	0.098–4.984
Smoke Yes	1.735	0.781–3.854
Alcohol Abstinent	1.395	0.650–2.995
Alcohol Weekend	1.441	0.570–3.641
Physical Activity No	0.495	0.251–0.978

The independent variables include continuous job demand score, continuous job control score, continuous job support score, age, duration of employment, BMI, SBP, DBP, educational level, alcohol intake, physical activity, and smoking habit.

A high score in work control and work support showed a positive effect on well-being, while older age and physical inactivity showed a negative effect (Table 5).

The median predicted CVD risk scores in the four Karasek categories and the two job support categories, based on the linear regression model, are shown in Table 6.

**Table 6:** Predicted median CVD risk scores in the four Karasek’s categories and the two job support categories, as per the linear regression model outlined in Table 4.

Karasek’s categories	Low job support median predicted CVD risk (IQR)	High job support median predicted CVD risk (IQR)
Passive	10.18 (7.28–11.94)	8.93 (5,78–11.59)
Low Strain	-	-
Active	-	-
High strain	10.29 (7.39–12.93)	8.44 (6.85–12.14)

## 4 Discussion

In line with previous studies, our survey suggests a correlation between work support and lower CVD risk (36). Our results support the hypothesis that certain dimensions of work-related stress may have an impact on CVD risk (37). Specifically, modifiable and non-modifiable risk factors may contribute to an increased likelihood of developing CVD and experiencing decreased well-being in work environments where peer support is lacking (38, 39). In contrast, general well-being and work support have been reported to increase long-term survival rates among individuals with chronic heart disease (40).

Based on Karasek’s classical dimensions, when analysing the levels of work-related stress, the study population showed a three-quarter distribution represented by high-stress job categories. However, compared to similar studies conducted on mixed populations using the same assessment method, the median scores for job demands and job control in our logistics support company were higher (14 vs. 12 for job demands; 23 vs. 18 for job control). It is worth noting that high-stress jobs have been consistently associated with detrimental health outcomes, such as cardiac autonomy imbalance indicated by Heart Rate Variability, hypertension and metabolic syndrome (41–43). In our study population, we observed an impact of job stress on perceived well-being, but not statistically significant on CVD risk. It is conceivable that working in a company that provides distribution support does not involve physically demanding tasks that significantly influence the perception of job stress (44). With regard to the other known risk factors for CVD, our results are in line with previous reports showing a protective effect of education (35, 45). Education is often used as a surrogate for socioeconomic status in epidemiological studies, which in turn implies a higher likelihood of performing high-stress, low-control and low-support jobs (46).

Our results are also consistent with previous research indicating a possible influence of work-related stress dimensions on psychological well-being, independent of other potential confounders such as shift work. In fact, night shifts could lead to alterations in circadian rhythms with an increased risk of developing sleep disorders and psychiatric disorders (47, 48). However, it is possible that other factors not considered, such as domestic stress or job-specific factors (e.g., security personnel), influenced our results (49). In our study population, the prevalence of current smokers (30%) and overweight individuals (55.6%) exceeded the national averages in Italy (19.3 and 54.8%, respectively) (50, 51). We also observed a clear protective role of regular recreational physical activity in reducing the risk of CVD. Therefore, the implementation of health promotion strategies aimed at physical activity and smoking cessation in these workers could contribute to improved well-being and productivity (52, 53). Several workplaces have successfully introduced health promotion strategies focusing on physical activity and smoking cessation and achieved significant cardiovascular risk reduction (54–58). In our study population, the average probability of experiencing a major cardiovascular event within 10 years was 6.9 per cent. After suffering a major cardiovascular event, occupational physicians must consider the economic and social consequences for workers, including the ability to return to work, fitness for work, loss of productivity and loss of job skills (59). Simple CVD risk calculation tools, such as the one used in our study, can help occupational physicians identify otherwise healthy workers who may be susceptible to developing CVD during routine workplace health surveillance. These workers may in fact not feel the need to consult a medical specialist. Finally, the average age of our study population was 43.27 years (sd 13.23), suggesting a significant trend towards an ageing workforce, even in the logistics support sector. The ageing of the workforce is a shared reality in most developed economies and can have a negative impact on labour productivity (60). Consequently, active ageing has become a major concern in occupational medicine and public health (61, 62). It is important to recognise certain limitations that affect the interpretation of our results.

The cross-sectional design of our study inherently prevents drawing firm conclusions. In discussing the limitations of a study focused on a single logistics company, there are main concerns regarding the generalisability of the results. The applicability to different sectors suggests that the results obtained from a specific company in the logistics sector may not be directly applicable to other sectors or work contexts. Each sector has its own unique dynamics, work stresses, and working conditions that may affect worker well-being and health risks differently. Without a critical examination of how these results would translate to different contexts, the extent of their applicability remains uncertain. In addition, focusing on a single company raises questions about the representativeness of the study within the logistics sector as a whole. Working practices, company culture, and stress factors may vary significantly between companies within the same sector. This study may therefore reflect the peculiarities of that company’s specific working environment rather than providing a general picture applicable to all logistics companies.

To address these limitations, it would be useful to conduct comparative studies that include a wider range of companies within the logistics sector and, ideally, studies that examine different sectors to assess the consistency of the observed effects. In addition, subgroup analysis within the current study could provide insights into how various factors (such as type of work, age of workers, or level of education) influence the relationship between working conditions and worker well-being. These steps could improve the generalisability and applicability of the results to broader contexts.

Furthermore, we could not assess the role of stress and social support at home, which might interact with work-related stress to increase cardiovascular risk. Despite these limitations, an advantage of our study was to focus on the general mental and physical health status of the working population by combining the assessment of work-related stress with that of CVD risk. Adopting such a holistic approach allows occupational physicians to address modifiable factors, such as work stress management through work organisation interventions, as well as lifestyle factors. The current research contributes to the investigation of the intricate relationship between the theoretical frameworks that predict CVD risk and any observed early changes that precede disease manifestation to establish preventive strategies. Our findings propose that various dimensions of work-related stress have the potential to influence both CVD risk and the psychological well-being of individuals in the workforce. Modifiable factors such as coworker support, decision-making autonomy, nature of work performed, and shift work schedules should be considered when attempting to mitigate CVD risk through interventions targeting organisational stress and promoting healthy lifestyles. In recent years, the ageing of the workforce is a multifaceted phenomenon that requires careful consideration and new proactive strategies such as prevention campaigns for smoking, alcohol abuse and proper nutrition (63, 64). Occupational physician must adapt their practice to meet the health needs of workers at different stages of their careers. By embracing age diversity and implementing prevention policies, companies can capitalise on the strengths of a multi-generational workforce, fostering a culture of inclusion and innovation.

In addition to domestic stress and shift work, there may be numerous other unmeasured or unconsidered factors that could influence the results, such as pre-existing health conditions, lifestyle (e.g., diet, physical activity), and social support. Domestic stress, whether stemming from family responsibilities, financial concerns, or interpersonal conflicts, can take a toll on one’s cardiovascular well-being. Research indicates that chronic stress contributes to hypertension, inflammation, and other risk factors for heart disease (64). Employees with pre-existing health conditions, such as hypertension, diabetes, or obesity, face additional hurdles in maintaining cardiovascular health in the workplace. Healthy lifestyle choices, including balanced nutrition, regular sleep cycle and regular physical activity, play a pivotal role in preventing cardiovascular disease (65). Yet, busy work schedules and sedentary office environments often present barriers to maintaining these habits (66). Finally, social support plays a critical role in mitigating the impact of stress and promoting cardiovascular health. Strong support networks in the workplace can buffer against the negative effects of stress, providing individuals with resources and encouragement to cope effectively. Employers can facilitate social connections among employees through team-building activities, mentorship programs, and open communication channels. By fostering a supportive and inclusive work environment, employers can contribute to the overall well-being of their workforce and reduce the burden of cardiovascular disease (66). The lack of control for these factors may introduce bias into the results and limit the ability to attribute observed effects with certainty to the variables of interest studied. To overcome these limitations, future studies should consider adopting a more holistic approach that includes a wide range of potential confounders and utilises methods of data collection and statistical analysis capable of handling the complexity of interactions between various factors. This would improve the internal validity of the study and the relevance of its findings, providing a more complete and accurate understanding of the impact of job stress and other factors on worker well-being. In conclusion, further investigation, using a longitudinal study design but also correlation with biohumoral parameters as done in other studies, will be indispensable to affirm the hypothesis that specific indicators of work-related stress and general well-being may predict susceptibility to the development of cardiovascular diseases (67).

## Data availability statement

The original contributions presented in the study are included in the article/supplementary material, further inquiries can be directed to the corresponding author.

## Ethics statement

Ethical approval was not required for the studies involving humans because Since the normal diagnostic care pathway was not changed, no therapies or drugs were administered, ethics committee approval was not required. The studies were conducted in accordance with the local legislation and institutional requirements. The participants provided their written informed consent to participate in this study.

## Author contributions

SF: Data curation, Formal analysis, Investigation, Resources, Supervision, Validation, Visualization, Writing – original draft, Writing – review & editing. GM: Conceptualization, Data curation, Formal analysis, Investigation, Supervision, Writing – review & editing. MV: Data curation, Supervision, Validation, Visualization, Writing – review & editing. AC: Resources, Supervision, Validation, Visualization, Writing – review & editing. DM: Formal analysis, Supervision, Validation, Visualization, Writing – review & editing. EC: Conceptualization, Data curation, Formal analysis, Funding acquisition, Investigation, Methodology, Project administration, Resources, Software, Supervision, Validation, Visualization, Writing – original draft, Writing – review & editing.
